# Organotropism: new insights into molecular mechanisms of breast cancer metastasis

**DOI:** 10.1038/s41698-018-0047-0

**Published:** 2018-02-16

**Authors:** Wenjing Chen, Andrew D. Hoffmann, Huiping Liu, Xia Liu

**Affiliations:** 1 Department of Pharmacology, Northwestern University, Chicago, IL USA; 2 Department of Medicine, Division of Hematology and Oncology, Northwestern University, Chicago, IL USA; 3 Robert H. Lurie Comprehensive Cancer Center, Northwestern University, Chicago, IL USA

## Abstract

Metastasis accounts for 90% of breast cancer mortality. Despite the significant progress made over the past decade in cancer medicine our understanding of metastasis remains limited, therefore preventing and targeting metastasis is not yet possible. Breast cancer cells preferentially metastasize to specific organs, known as “organotropic metastasis”, which is regulated by subtypes of breast cancer, host organ microenvironment, and cancer cells-organ interactions. The cross-talk between cancer cells and host organs facilitates the formation of the premetastatic niche and is augmented by factors released from cancer cells prior to the cancer cells’ arrival at the host organ. Moreover, host microenvironment and specific organ structure influence metastatic niche formation and interactions between cancer cells and local resident cells, regulating the survival of cancer cells and formation of metastatic lesions. Understanding the molecular mechanisms of organotropic metastasis is essential for biomarker-based prediction and prognosis, development of innovative therapeutic strategy, and eventual improvement of patient outcomes. In this review, we summarize the molecular mechanisms of breast cancer organotropic metastasis by focusing on tumor cell molecular alterations, stemness features, and cross-talk with the host environment. In addition, we also update some new progresses on our understanding about genetic and epigenetic alterations, exosomes, microRNAs, circulating tumor cells and immune response in breast cancer organotropic metastasis.

## Introduction

Breast cancer remains the most common malignancy in women. About 20 to 30% of patients with early-stage breast cancer will experience distant metastases. Approximately 90% of patient deaths are because of complications from recurrent or metastatic diseases.^1^ Distant metastasis is a complex multistep process. Tumor cells must detach from the primary tumor and intravasate into systemic circulation, survive in the circulation, evade immune attacks, adhere to the capillaries and extravasate before they colonize distant organs.^1^

The distribution of distant metastases to certain organs is a non-random process known as “metastatic organotropism”,^2^ which is regulated by multiple factors such as subtypes of cancer, molecular features of cancer cells, host immune microenvironment, and cross-talk and interactions with local cells. Host microenvironment can be modified to form a pre-metastatic niche (PMN), a supportive environment for tumor growth in a host tissue before a tumor spreads. PMN is regulated by tumor cell-secreted factors and exosomes, non-resident cell recruitment, and host cell alternations.^3^ Tumor cells can also interact with extracellular matrix (ECM) of host tissue to facilitate metastasis. Steven Paget^4^ proposed the “seed and soil” theory in 1889 to described the site-specific metastasis. The ability of tumor cells to initiate growth largely depends on cross-talk between metastatic tumor cells (“seed”) and host microenvironment (“soil”). In addition, organotropic metastasis is driven by different barriers of the host organ, including specific functions of the organ and limitations on how the cancer cells breach the barrier in order to extravasate to distinct distant organs. For example, the capillary endothelia are backed by a basement membrane in the lungs between lung alveoli and pulmonary capillaries to allow gas exchange at the blood-air barrier. In the brain these capillary endothelia are strengthened by tight junction proteins and astrocyte foot processes in the blood–brain barrier (BBB).^5^ Whereas in liver and bone marrow, fenestrated sinusoidal endothelia usually have a higher permeability to facilitate large molecule transport.^6^ In this review, we are focusing on the cellular and molecular mechanisms of breast cancer organotropic metastasis, including tumor cell intrinsic features and cross-talk with host environment.

## Multiple factors determine the organotropic metastasis of breast cancer

### Subtype-dependent metastasis organotropism

Histologically, breast cancer is broadly categorized into in situ carcinoma and invasive (or infiltrating) carcinoma, and most breast cancers are invasive. More than 80% of the invasive breast cancers are invasive ductal carcinomas (IDCs), and the rest are invasive lobular carcinomas (ILCs).^7^ The organ preference of metastasis in ILC and IDC is distinct. IDCs tends to metastasize to lungs, distant lymph-nodes and central nervous system (CNS), whereas ILC has three times more metastases in the peritoneum, gastrointestinal tract, and ovaries.^8^

However, studies focused on tumor cell biology have shown that histological differences are not sufficient prognostic markers for metastasis risk if being utilized alone, without biological markers.^7^ Biological markers classify breast cancers into molecular subtypes. These markers are analyzed by immunohistochemical staining or microarray-based gene expression as the newly developed prediction analysis of microarray of 50-gene set (PAM50). Examples are hormone receptors (HRs) including estrogen receptor (ER) and progesterone receptor (PR), human epidermal growth factor receptor 2 (HER2), the cell proliferation marker Ki67, cytokeratin 5/6 (CK5/6), and epidermal growth factor receptor (EGFR). Based on these markers, breast cancer molecular subtypes are classified as follows: luminal A (ER-positive and/or PR-positive, HER2-negative and Ki67 low), luminal B (ER-positive and/or PR-positive, HER2-negative and Ki67 high), luminal-HER2 (ER-positive and/or PR-positive and HER2-positive), HER2-enriched (ER-negative, PR-negative, HER2-positive), basal-like (ER-negative, PR-negative, HER2-negative, and EFGR-positive or CK5/6-positive), and triple-negative phenotype (TN) (ER-negative, PR-negative, HER2-negative). TN tumors have a high frequency of p53 mutations and 80% of them express basal-like phenotype. TN tumors negative for both EGFR and CK5/6 are labeled TN-non basal (Table 1).^9^

**Table 1:** Breast cancer molecular subtypes and median duration of survival with distant metastasis

Molecular subtypes	Biological markers	Median durations of survival from time of first distant metastasis
Luminal A	ER-positive and/or PgR-positive, HER2-negative and Ki67low	2.2 years
Luminal B	ER-positive and/or PgR-positive, HER2-negative and Ki67high	1.6 years
Luminal-HER2	ER-positive and/or PgR-positive and HER2-positive	1.3 years
HER2-enriched	ER-negative, PgR-negative, HER2-positive	0.7 years
Basal-like	ER-negative, PgR-negative, HER2-negative, and EGFR-positive or CK5/6-positive	0.5 years
Triple-negative phenotype (TN)	ER-negative, PgR-negative, HER2-negative, high frequency of p53 mutations. 80% of them express basal-like phenotype, and negative for both EGFR and CK5/6 are called TN-nonbasal	0.9 years

See ref.^16^

**Fig. 1:**
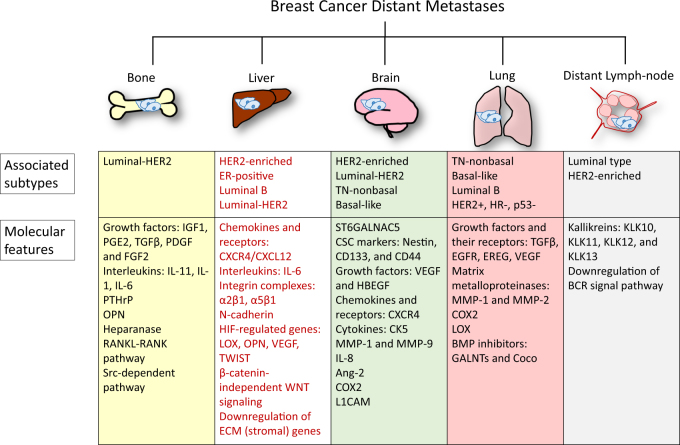
Summary of breast cancer organotropic metastases. The site-specific organotropic metastasis is regulated by the breast cancer subtypes, different gene signatures and signaling pathways of metastatic tumor cells. Bone is the most common site of metastatic breast cancer patients, with the second most common site is brain, and liver and lungs are the next

Besides locoregional relapse, breast cancer tends to metastasize distantly to the bone, brain, liver, lung, and distant lymph-nodes.^10^ The most common distant metastasis is the bone which occurs in 70% of metastatic breast cancer patients.^11^ The next most common site of metastases is the liver, at an approximate rate of 30%,^12^ and the brain is the third most common site around 10–30%.^13^ Different breast cancer subtypes correlate with significantly distinct overall survival and differing tendencies to metastasize to specific organs (Fig. 1).^14^ A recent study by the Surveillance, Epidemiology, and End Results Program evaluated the relationship between breast cancer subtypes and sites of distant metastasis.^15^ The results demonstrate that all subtypes are prone to bone metastases, especially the HR^+^/HER2^+^ (luminal-HER2) subtypes. The HR^−^/HER2^+^ (HER2-enriched) subtype has a higher probability of brain metastasis compared to the HR^+^/HER2^−^ (luminal A and luminal B) subtype. The HER2-enriched subtype also has more liver metastases compared to the HER2-subtypes. Patients with TN breast cancer primarily present lung metastases. In a multivariate analysis comparing different subtypes, luminal-HER2 and HER2-enriched subtypes show a significantly higher rate of metastases to brain, liver, and lung than luminal A HER2-negative subtype. Both basal-like and other TN subtypes have a high rate of metastases to brain, lung, and distant lymph-node. However, basal-like subtype is specifically associated with a low rate of liver and bone metastases.^16^ Other studies also showed the preferential of molecular subtype-based organotropic metastasis, which will be discussed in each individual section and is summarized in Fig. 1.

### Genetic alterations and gene expression features of organotropic metastasis

Next-generation DNA sequencing and transcriptome analysis have brought breakthrough discoveries to facilitate precision medicine in cancer metastasis. During tumor development and progression, cancer cells accumulate genetic mutations, which may converge into alternations of important genes and pathways. A recent large-scale genomic evolution study of patients with breast cancer metastasis and local relapse showed a higher mutation burden in metastases than that of primary tumors, such as inactivation of SWItch/sucrose non-fermentable, and Janus kinase 2-signal transducer and activator of transcription 3 (JAK2-STAT3) pathways.^10^ In another study, the genetic alterations identified in the liver metastases of various cancers include mutations/chromosomal inversions of Notch pathway genes, mutations/rearrangements in the fragile histidine triad gene which regulates purine metabolism, and other shared mutations in genes impact the immune response to metastatic cells.^17^ Therefore, organ-specific metastases from different primary cancer types may bear common genetic aberrations in order to adapt to the same distant immune and host metabolic microenvironment.

The tissue-specific gene signatures and signaling pathways have been identified by comparing the tumor cells in the primary site to the distant lesions in the organ of interest in breast cancer animal models. These molecular features predict the metastatic organotropism of circulating tumor cells (CTCs). In general, the transcriptome profiles of the bone and lung metastases are quite distinct with only a few genes in common. The most prominently overexpressed genes in bone metastasis identified by Kang et al.^18^ encode cell surface and secretory proteins, each of which may alter the bone tissue environment to foster formation of osteolytic bone lesions and have little overlap with the poor prognosis signatures identified by van't Veer et al.^19^ However, the signature genes identified by Minn et al.^20^ for lung metastasis are less specific to the lung microenvironment, rather promoting aggressive growth and invasiveness, and overlap more with poor prognosis genes.^19^ Some molecules could play context-dependent roles in different metastasis sites. For example, transforming growth factor beta (TGFβ) promotes metastasis of breast cancer to the lungs but it is dispensable to bone metastasis, which is mediated by Src-dependent signaling pathway.^21, 22^ Upon insulin-like growth factor (IGF1) stimulation, the bone-seeding cancer cells exhibit a greater level of IGF1 receptor (IGF1R) phosphorylation than the brain-homing cancer cells.^23^ EGFR ligands and cyclooxygenase 2 (COX2) are associated with metastases to the lungs, but not to the bone or liver.^20^ Breast cancer cell-secreted Dickkopf-1 also has differential effects on the metastases to the lungs and bone. It suppresses lung metastasis by antagonizing non-canonical Wnt-signaling pathway but promotes bone metastasis by regulating canonical Wnt-signaling of osteoblasts.^24^ Hence, the formation of metastases is finely tuned by many signaling pathways of tumor cells and their cross-talk with host organs. We will discuss organ-specific molecular features in more details in following sections.

### Exosomes, microRNAs (miRs) and stemness in organotropic metastasis

Exosomes are small membrane-bound vesicles (30–100 nm) containing functional biomolecules (including proteins, RNA, DNA and lipids) that can be horizontally transferred to recipient cells.^25^ Accumulating evidences suggest that exosomes play a critical role in organ-specific metastasis. For example, brain astrocyte-derived exosomes can promote the outgrowth of brain metastatic cancer cells by transferring PTEN-targeting miR-19a to these cancer cells.^26^ Lyden et al.^27^ demonstrated that the exosomes derived from organotropic metastatic cancer cells can be preferentially up-taken by specific host organ cells to prepare the PMN. Exosome proteomics identify distinct expression patterns for organotropic metastases of breast cancer cells, in which integrin αvβ5 of cancer exosomes specifically binds to Kupffer cells, facilitating liver metastasis, whereas exosomal integrins α6β4 and α6β1 bind lung-resident fibroblasts and epithelial cells, preparing lung metastasis. Targeting the integrins α6β4 and αvβ5 decreases exosome uptake, as well as lung and liver metastasis, respectively. They also indicated that exosomal integrins are endocytosed by organ-specific resident cells to establish PMN via Src activation and pro-inflammatory S100 expression.^27^

MiRs are small noncoding RNAs that play a central role as master regulators of gene expression in multiple cancer-related signaling pathways. The role of miRNAs in metastasis was first reported by the Weinberg group.^28^ They found that overexpression of miR-10b in non-metastatic breast tumors can initiate robust invasion and metastasis by increasing expression of a pro-metastatic gene RHOC (Ras homolog gene family, member C). Recent studies suggested that miRs regulate organotropic metastasis by reprogramming PMN, targeting host microenvironments and regulating cancer stem cell (CSC) functions. For example, miR-122 promotes breast cancer metastasis to the brain and lungs by reprogramming glucose metabolism in the PMN.^29^ Expression of the miR-23b/27b/24 cluster promotes lung metastasis by targeting metastasis-suppressive gene prosaposin, which negatively correlates with metastatic progression in breast cancer patients.^30^ In endothelial monolayers, exosome-mediated transfer of metastatic breast cancer cell-secreted miR-105 destroys vascular endothelial barriers by targeting the tight junction protein Zonula occludens (ZO-1), therefore promoting lung and brain metastasis.^31^ This finding suggests that exosomes can either directly regulate or function as vehicle to deliver molecules including miRs to promote organ-specific metastasis.

In addition, miRNAs regulate the capacity of CSCs to regulate metastasis. MiR-30c and its family members are associated with favorable distant metastasis-free survival of breast cancer patients, likely through targeting epithelial-to-mesenchymal transition mediator TWF1, and thereby inhibiting CSC-mediated lung metastasis and chemotherapy-resistance.^32^ CSC populations of CD24^−^/CD44^+^/ESA^+^ cells isolated from metastatic breast cell lines are highly metastatic to bone and brain, and express significantly lower levels of miR-7, which attenuates the invasion and self-renewal capabilities of CSCs by modulating KLF4.^33^ In contrast, miR-495 is upregulated by transcription factors E12/E47 in different CSC subpopulations (PROCR^+^/ESA^+^ and CD44^+^/CD24^−/low^), and promotes oncogenesis and hypoxia resistance via downregulation of E-cadherin and DNA damage response 1 (REDD1).^34^ MiR-199a can promote both normal and cancerous mammary stem cell properties by repressing nuclear receptor corepressor LCOR, which primes interferon (IFN) responses.^35^ Further studies suggest that the miR-199a–LCOR axis is activated in poorly differentiated ER-breast cancer to promote tumor initiation and metastasis by maintaining CSC self-renewal competence and avoiding differentiation or senescence induced by suppressive immune cytokine IFN-α.^35^ Recently, in order to identify miR signatures of organotropic breast cancer metastasis, Schirijver et al.^36^ compared global miR expression in 23 primary breast cancer specimens with their corresponding multiple distant metastases. They found that miR-106b-5p is an independent predictor of lung and gastrointestinal metastases, miR-7-5p of skin metastases and miR-1273g-3p of ovarian metastases, suggesting that miR signatures can be used to predict metastatic organotropism.

### CTCs and circulating CSCs in organotropic metastasis

The spreading tumor cells in the blood circulation, or CTCs, are considered the “seeds of metastasis”.^37^ To metastasize, these CTCs must acquire the capacity to colonize distant organs. Unlike white blood cells which can recirculate to the site that is conductive to adhesion, most CTCs are passively entrapped in the first capillary bed they encounter. Breast cancer cells escape from the primary site into the blood circulation are carried by the blood flow through the heart, and then to the capillary beds of the lungs, where many of these CTCs will arrest. Some CTCs might pass through the lung to enter the systemic arterial system, where they are transported to capillary beds in other organs, such as bone.^38^ The organotropic metastasis of cancer cells is regulated by the survival of CTCs in the blood circulation, the ability of CTCs to adhere to the endothelium, the blood flow pattern and the physical vascular architecture of the metastatic site, and the microenvironment of the “soil” to favor growth of secondary tumors.^39^ It is well accepted that the initial arrest of CTCs to specific organs is primarily mechanical, however, their subsequent growth will depend on the compatibility of the “seed” with the “soil” in the new organ. Therefore, molecular analyses of CTCs are important to understand the role of CTCs in organotropic metastasis, and develop therapeutics to targeting CTCs.

Several studies have shown the presence of stem cell-like markers in CTCs, leading to the hypothesis that circulating cancer stem cells (CCSCs) represent a distinct subpopulation of CTCs that bear metastasis-initiating capabilities to disseminate and colonize in distant organs.^40^ CCSCs isolated from luminal breast cancer patients express epithelial cell adhesion molecule EpCAM, CD44 and CD47 and initiate metastasis to the bone, lung and liver in mice.^37^ Sihto et al.^41^ demonstrate that tumors from patients with the brain as the first metastatic site are negative for ER and PR but frequently expressed CK5 and CSC markers nestin and CD133. Similarly, a CD133^hi^CD44^hi^ subpopulation of the TN breast cancer cell line GI-101 develop significantly more brain metastases in mouse models.^42^ Within the circulatory system, CTCs have to protect themselves from various host attacks, such as immune assaults, apoptosis and shear stress to survive. Previous studies have highlighted that platelets not only guard the CTCs from immune elimination but also promote their arrest at the endothelium, extravasation and establishment of secondary lesions.^43^ Platelets can rapidly coat the CTCs after they enter the blood stream, then prevent the recognition and lysis by natural killer (NK) cells through releasing TGFβ and platelet-derived growth factor (PDGF) that inhibit NK cell activity.^44^ TGFβ and PDGF are both highly correlated with breast cancer bone and lung metastases, and PDGF receptor-α expression in breast cancer is associated with lymph-node metastasis.^45^ In the meantime, platelet-derived TGFβ can also induce epithelial–mesenchymal transition (EMT) of CTCs.^46^ Platelet-derived Autotaxin (ATX) cooperates with secreted lysophosphatidic acid (LPA) upon CTC-induced platelet activation to promote skeletal metastasis of breast cancer.^47^ The precursor cells of platelets megakaryocytes also promote breast cancer metastasis to the bone by assisting the extravasation of CTCs to the bone marrow.^48^

### The immune system in organotropic metastasis

The immune system contributes to each cascade of metastasis. At the primary site, it is involved with PMN formation in specific sites. For example, in a xenograft model of the human breast cancer cell line MDA-MB-231, tumor cells induce CD11b^+^ immune suppressor myeloid cells recruitment in the pre-metastatic lung via secretion of lysyl oxidase (LOX).^49^ Primary breast tumor hypoxia can also induce CD11b^+^/Ly6C^med^/Ly6G^+^ myeloid cell accumulation and reduces the NK cell cytotoxicity in the pre-metastatic lung.^50^ Moreover, recruitment of functional monocytes/macrophages by tissue factor-mediated coagulation is essential for metastatic cell survival and PMN establishment in the lungs.^51^ When tumor cells enter the circulation, immune cells also interact with tumor cells and affect the metastatic sites. Studies have suggested that neutrophils can assist metastasis of CTCs. In response to inflammatory cues, neutrophils release neutrophil extracellular traps (NETs) which can capture CTCs and support the formation of micrometastases.^52^ Metastatic breast cancer cells also induce neutrophils to make metastasis-promoting NETs and support lung colonization.^53^ However, tumor-entrained neutrophils inhibit metastatic tumor cell seeding to the lungs by generating H_2_O_2_, upon activation by tumor secreted CCL2 (chemokine ligand 2).^54^ The neutrophil polarization (N1 vs. N2) which is regulated by specific tumor-derived factors such as TGFβ may explain these inconsistent results, however when and where neutrophil polarization is shaped remains to be elucidated.^55^ In lung cancer studies, neutrophils promote liver metastasis via neutrophil macrophage-1 (Mac-1) mediated interaction with intercellular adhesion molecule 1 in CTCs,^56^ and interactions between adherent neutrophils and CTCs within the inflamed liver sinusoids may further increase tumor cell arrest in the liver.^57^ However, whether neutrophils play the similar roles in breast cancer metastasis warrants further investigation.

T cells also participate in regulation of organotropic metastasis by expressing different proteins. A study has shown that IL-17-producing gamma delta (γδ) T cells activate the expansion and polarization of neutrophils which in turn suppress cytotoxic CD8^+^ T cells and promote lung metastases.^58^ T cell-expressed prolyl-hydroxylase proteins can create immunoregulatory environment for lung, thus facilitating tumor cell colonization and metastasis formation by limiting pulmonary type helper (Th)-1 responses, promoting T_reg_ cell induction, and restraining CD8^+^ T cell effector function.^59^ Moreover, CCR4 expressing T_reg_ cells are required for lung metastasis by directly eliminating tumor suppressing NK cells through beta-galactoside-binding protein.^60^ During bone metastasis of breast cancer, tumor-specific RANKL expressing T cells induce pre-metastatic osteolytic bone disease and promote bone metastasis formation.^61^

Interactions between immune cells, host environment and tumor cells are essential for the organ-specific metastasis formation. For breast cancer lung metastasis, breast tumor-evoked regulatory B cells promote lung metastasis by converting resting CD4^+^ T cells to Treg cells, which perform immune suppression role.^62^ Depletion of the host sphingosine-1-phosphate transporter spinster homolog 2 (Spns2) can increase the infiltration of effector T cells and NK cells into the lung, and reduce TN breast cancer cell line lung colonization and melanoma cell line lung metastasis.^63^ In addition, blocking human M2 macrophage differentiation by COX2 inhibitor reduced lung metastasis.^64^ For breast cancer bone metastasis, both clinical data and mouse model showed that silencing of IFN regulatory factor Irf7 pathways in breast cancer promotes bone metastasis through innate immune escape.^65^ Depletion of plasmacytoid dendritic cells inhibits tumor growth and prevents bone metastasis by activating tumor-specific cytolytic CD8^+^ T cells.^66^

## Bone metastasis

Bone is the most common site of metastatic breast cancer and accounts for about 70% of metastases.^11^ It is frequently associated with osteolytic type metastatic lesions due to hyperactive osteoclast-mediated bone resorption.^67^ Although all the subtypes are prone to bone metastasis, luminal subtype tumors develop bone metastasis at a much higher rate (80.5%) than basal-like (41.7%) and HER2-like tumors (55.6%).^68^

### Bone metastasis molecular features

Integrin complexes play important roles in bone metastasis of breast cancer. Study showed that integrin αvβ3 overexpression in tumor cells promotes metastasis to bone by mediating tumor cell adhesion and signal transmission for tumor progression.^69^ Fully activated integrin αvβ3 is required in the process of LPA production, which can be induced by ATX and shows growth factor-like activities.^47^ Another integrin complex α4β1 is expressed in some osteoclast progenitors, which can promote osteolytic expansion of indolent bone micrometastasis to overt metastasis by interacting with vascular cell adhesion molecule 1.^70^ Moreover, Runt-related transcription factor 2 promotes the attraction and adhesion of breast cancer cells to the bone and confers cancer cell survival and bone colonization advantages in an integrin α5-dependent manner.^71^

Cytokines, chemokines and other growth factors can also promote bone metastasis formation. Among the genes elevating osteolytic metastatic activity, the prometastatic cytokine TGFβ can stimulate the expression of osteolytic angiogenic factors interleukin-11 (IL-11) and CTGF.^18^ SMAD4 is a tumor suppressor that inhibits tumor cell proliferation, however, it is also an osteolytic metastasis promoter linking TGFβ signaling to a subsequent induction of IL-11.^72^ Both hypoxia (via HIF-1α) and TGFβ signaling activate VEGF and the CXC chemokine receptor 4 (CXCR4) to drive breast cancer bone metastases.^73^ Human antigen R-regulated chemokine CCL20 promotes bone metastasis in basal-like TN breast cancer by elevating the secretion of matrix metalloproteinase (MMP)-2/9 and the ratio of receptor activator of nuclear factors kappa-B ligand (RANKL)/osteoprotegerin, which is critical in the “vicious cycle”.^74^ Thus, CCL20 may serve as a therapeutic target in breast cancer patients with bone metastasis.

Recently, a retrospective study reviewed the clinical characteristics and risk factors for bone metastases in breast cancer patients comparing to the patients without bone metastases. The results showed more axillary lymph-node metastases, high serum concentrations of cancer antigen (CA) 125, CA153, alkaline phosphatase and low level of hemoglobin are closely related to bone metastases.^75^ In order to understand the mechanisms underlying the development of distant metastases, Van de Vijver group analyzed gene expression signatures specifically associated with the development of bone metastases in breast cancer patients, and identified a 15-gene expression signature significantly correlated to the bone metastasis status of breast cancer.^65^ These 15 genes are APOPEC3B, ATL2, BBS1, C6orf61, C6orf167, MMS22L, KCNS1, MFAP3L, NIP7, NUP155, PALM2, PH-4, PGD5, SFT2D2 and STEAP3, which encode mainly membrane-bound molecules with molecular function of protein binding. The expression levels of the up-regulated genes (NAT1, BBS1 and PH-4) correlated with EMT status of the tumor.^65^

### Vicious cycle: cross-talk of tumor cells and bone microenvironment

**Fig. 2:**
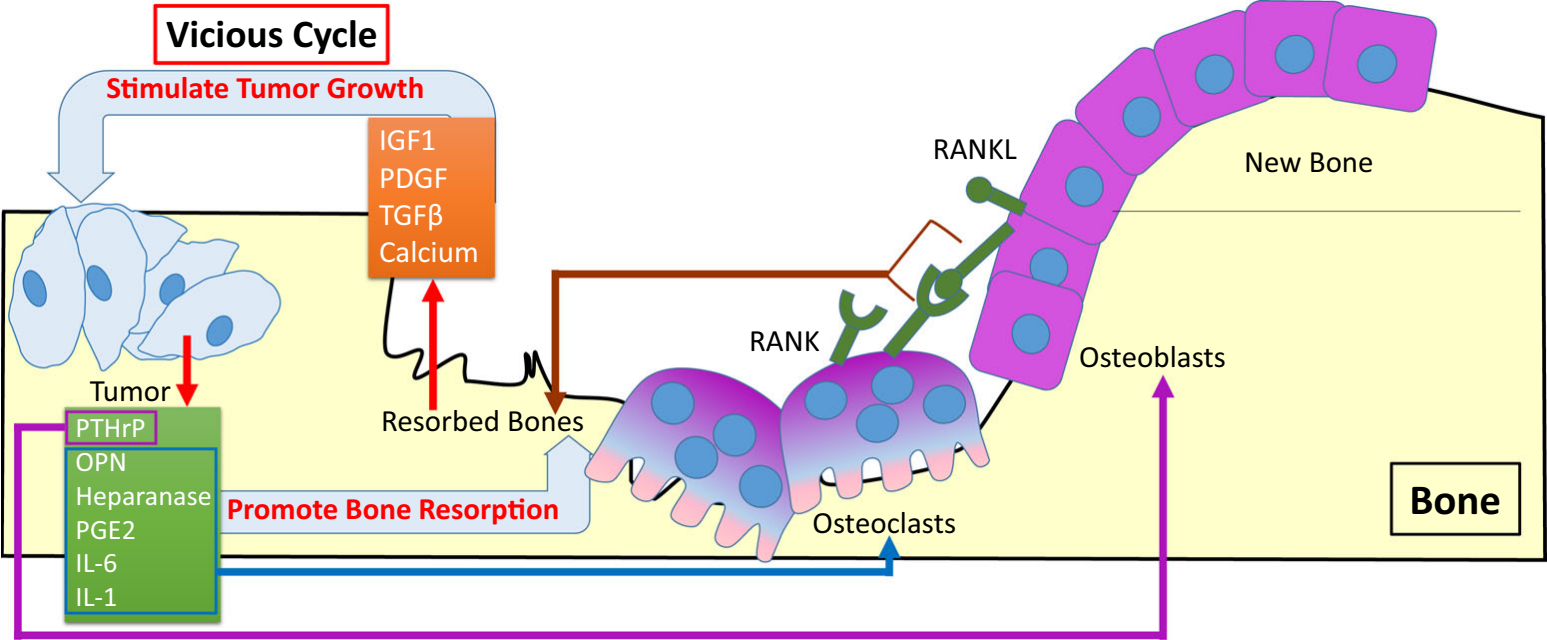
Vicious cycle of bone metastasis. Tumor-derived factors such as OPN, PTHrP, heparanase, IL-1, IL-6 and PGE2 enhance the osteoclasts formation and promote bone resorption.^77, 78^ Resorbed bones release bone-derived growth factors, such as IGF1, PDGF, and TGFβ, and calcium, which in turn stimulate tumor growth.^79^ Tumor cells that reach in the bone microenvironment secrete PTHrP to initiate osteolysis and stimulate bone lining osteoblasts.^80^ In response, the expression of RANKL is upregulated by activated osteoblasts and binds to its receptor RANK to activate RANKL-RANK signaling pathway, and leading to bone resorption^81^

Breast cancer metastases to bone leads to bone loss by promoting bone degradation and interfering with bone remodeling.^11^ Metastatic breast cancer cells extravasate from capillaries to the bone marrow and gain the bone cell-like properties by osteomimicry that improves homing, adhesion, proliferation and survival in the bone microenvironment.^76^ More importantly, the relationship between bone resorption and tumor growth forms the “vicious cycle” (Fig. 2).^77^ Tumor-derived factors such as osteopontin (OPN), parathyroid hormone-related peptide (PTHrP), heparanase, IL-1, IL-6 and prostaglandin E2 (PGE2) enhance the osteoclasts formation and promote bone resorption.^77, 78^ Resorbed bones release bone-derived growth factors, such as IGF1, PDGF, and TGFβ, as well as calcium that stimulates skeletal tumor proliferation.^79^ This vicious cycle accelerates bone loss and provides a fertile soil for tumor growth.^67^ Several components have been identified as master factors in this process. Tumor cells that reach in the bone microenvironment secrete PTHrP to initiate osteolysis and stimulate bone lining osteoblasts.^80^ In response, the expression of RANKL is upregulated by activated osteoblasts and binds to its receptor RANK to form RANKL-RANK signaling pathway, which is involved in activating the differentiation of preosteoclasts into activated osteoclasts, and leading to bone resorption.^81^ The activated osteoclasts subsequently degrade the bone matrix by releasing hydrogen ions to create strong acid, and also releasing proteinases such as the cathepsin-K (cat-K), MMP-9, and MMP-13.^82, 83^ Bone degraded by osteoclasts can release TGFβ, IGF1, and other growth factors stored in the bone matrix, and these growth factors in turn stimulate tumor growth and lead to increased levels of tumor derived PTHrP.^83^

## Brain/CNS metastasis

There are 10–30% of patients with metastatic breast cancer develop brain/ CNS metastases.^13, 42^ Several factors associated with the increased risk of brain metastases have been identified, including young age, poorly differentiated tumors, HER2-enriched, luminal-HER2, basal-like and TN breast cancer subtypes, and four or more metastatic lymph-nodes.^16, 84^ In most cases, brain metastasis is viewed as a late complication of disease, and happens after metastases have appeared systemically in the lung, liver, and/or bone for which few effective treatment options exist.^42^ The two main sources of brain metastases are adenocarcinomas of the lung or the breast.^12^ Brain metastases are not only associated with an extremely poor prognosis but also with neurological impairments by often affecting both cognitive and sensory functions.^42^

Brain metastasis from breast cancer show patterns of parenchymal brain metastasis or leptomeningeal metastasis. Parenchymal brain metastasis account for approximately 80% of all brain metastases.^85^ Metastases to the brain parenchyma are thought to be hematogenous in origin.^86^ The co-option of the breast cancer cells with host vascular tissues is essential for tumor cells growth.^42^ For leptomeningeal metastases, breast cancer is the most common solid tumor origin.^85^ Once the tumor cells reach the leptomeninges, they may spread via the cerebrospinal fluid.^86^

### Breaching of the blood–brain barrier (BBB)

**Fig. 3:**
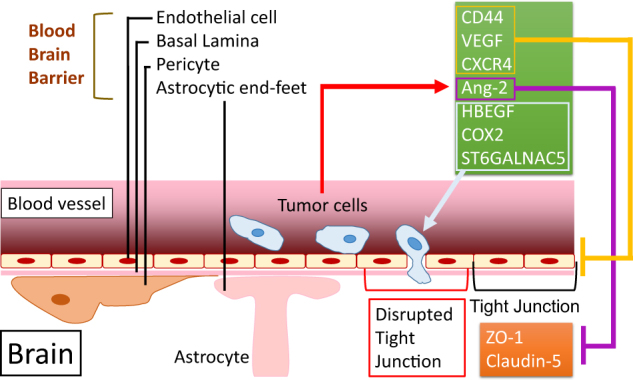
Brain metastatic cancer cells breach the blood–brain barrier (BBB). BBB is composed of capillary endothelial cells, basal lamina, pericytes and astrocytic end-feet.^88^ CD44, VEGF and CXCR4 can enhance the transendothelial migration of tumor cells by disrupting endothelial integrity.^90^ Ang-2 increases BBB permeability by impairing ZO-1 and Claudin-5 tight junction protein structures.^91^ COX2, HBEGF, and ST6GALNAC5 mediate cancer cell passage through the BBB^42, 87^

To form the brain metastasis, CTCs need to breach the interface between the circulation and the brain, the BBB, and then interact with local microenvironment in order to survive and then form the metastasis colony. Breaching the BBB involves mediators of extravasation through non-fenestrated capillaries, complemented by specific enhancers of BBB crossing and brain colonization (Fig. 3).^87^ BBB is composed of capillary endothelial cells backed up by basal lamina, pericytes and astrocytic end-feet.^88^ Tumor cells usually transmigrate the BBB through paracellular endothelial tight junctions.^89^ CD44, VEGF and CXCR4 can enhance this transendothelial migration process by disrupting endothelial integrity.^90^ Angiopoietin-2 (Ang-2) also increases BBB permeability by impairing ZO-1 and claudin-5 tight junctions protein structures and can cause the subsequent colonization of TN breast cancer cells in brain.^91^ Gene expression analyses of cells with high brain metastatic activity identified COX2, EGFR ligand heparin-binding EGF-like growth factor (HBEGF), and ST6GALNAC5 as mediators of cancer cell passage through the BBB.^87^ For example, ST6GALNAC5 specifically mediates brain metastases by enhancing tumor cells adhesion to brain endothelial cells.^92^ COX2 can promote the expression of MMP1, which is the only MMP significantly correlated with brain metastasis.^93^ Furthermore, COX2 and prostaglandin activate astrocytes to release chemokine (C–C motif) ligand 7, which in turn promotes self-renewal of CSCs or tumor-initiating cells in the brain.^93^ Moreover, the BBB is responsible for the breast cancer patients with brain metastases showing fewer CTCs compared with breast cancer patients with other metastases.^94^

### Brain metastasis molecular features

We have discussed that brain metastatic cells are related with some CSC markers, such as nestin, CD133^41^ and CD44.^42^ In comparisons of primary breast tumors with metastases, very high frequency of hypermethylated genes are found in metastases to the bone, brain, and lung.^95^ In particular, hypermethylation of cyclin D2, retinoic acid receptor-β, and hin-1 are more frequently detected in brain metastases.^86^ In addition to HER2, HER3 overexpression is also associated with brain metastases in breast cancer patients.^96^ The primary ligand of HER3/HER2 heterodimers heregulin (HRG) is highly expressed in the human brain and is able to induce the transendothelial migration of HER2/HER3-positive breast cancer cells across a tight barrier of brain microvascular endothelia. Finally, MMP-9 has been identified as one of the factors partially mediating this process.^97^ Interestingly, in breast cancer cells, HRG-induced MMP-1 and MMP-9 expression is mediated through HER3-dependent pathway and cells with higher HER2 level is more aggressive than those with the lower HER2 expression.^98^ A potential signature of brain metastasis marker HER2^+^/EGFR^+^/HPSE^+^/Notch1^+^ in EpCAM-negative CTCs has been identified as high invasive and capable of generating brain and lung metastases in xenograft model.^99^

### Interaction between brain metastatic cells and host cells

After tumor cells that have infiltrated into the brain in order to grow and develop into a metastatic lesion, they need to recruit blood vessels and establish the suitable metastatic microenvironment.^42^ Brain-seeking metastatic cells secrete significantly more VEGFA and IL-8 than the parental cells. VEGF is a principle angiogenic factor and contributes to the outgrowth of the brain metastases.^100^

When tumor cells arrive in the brain, there is an intensive cross talk with the residential brain cells. The association between brain microvascular cells, astrocytes and neurons forms functional “neurovascular units”, and recent studies have highlighted the importance of brain endothelial cells in this modular organization. Interactions between the brain endothelium, astrocytes and neurons that may also regulate BBB functions.^101^ In breast cancer, the brain metastatic cells gain the ability to exploit the brain endogenous substrates secreted by the resident cells to facilitate the oncogenic growth.^42^ Tumor cells may show the gamma-aminobutyric acid (GABA)-ergic phenotype as neuronal cells with upregulated proliferation by taking up and catabolizing GABA into succinate and subsequent NADH as biosynthetic source.^102^ Studies have shown that among different glial cells, astrocytes and microglia are associated with brain metastases. Local astrocytes can be activated by tumor cells^103^ and then secrete a host of soluble proteins including IL-1, IL-3, IL-6, IFNγ, tumor necrosis factor-α (TNF-α), TGFβ, IGF1, PDF1, and other cytokines.^104^ Many of these factors, such as IL-6 and TGFβ, can function as oncogenic signals for the tumor cells.^105^ In contrast, Plasmin from the reactive brain stroma inhibits metastatic invasion by converting membrane-bound astrocytic Fas ligand into a paracrine death signal for cancer cells and inactivating the neuronal cell adhesion molecule L1 cell adhesion molecule, which promotes the spread of tumor cells and formation of large metastases. To counter the inhibitory signals, tumor cells express high levels of anti-plasminogen activator serpins, including neuroserpin and serpin B2, to promote cancer cell survival and vascular co-option in brain metastasis.^106^ Co-cultured breast cancer cell lines with astrocytes exhibited astrocytes-derived factors MMP-2 and MMP-9, which induce both the migration and invasion of breast cancer cells.^104^ Microglia can also be activated by tumor cells and perform similar functions as astrocytes to promote colonization tumor cells, and this process occurs in a Wnt-dependent manner.^107^

## Lung metastasis

Compared to other subtypes, basal-like and luminal B subtypes of breast cancer are more aggressive and show higher levels of lung-specific metastasis.^84^ A new triple negative, p53 negative subtype is highly associated with lung metastasis in invasive ductal breast cancers.^108^ Compared to other metastatic lesions, lung metastatic cells have fewer roles in the lung microenvironment, but generally show aggressive growth and invasiveness.^109^

### Lung metastasis molecular features

Many lung metastasis signature (LMS) genes are associated with poor prognosis.^109^ From clinical data, patients with LMS-expressing primary tumors are associated with primary tumor growth and high risk of metastasis and therefore exhibit worse overall survival.^87, 110^ Genes such as epidermal growth factor receptor ligand epiregulin, COX2, MMP-1 and MMP-2 have been found to be associated with lung metastases by facilitating the angiogenesis in the tumor, releasing tumor cells into the circulation and breaching lung capillaries.^111^ Consistently, inhibition of EGFR and COX2 minimizes lung metastasis.^112^ Studies also show that protein deacetylase SIRT7 antagonizes TGFβ signaling and inhibits breast cancer lung metastasis.^113^

Lung metastasis formation also involves CSC functions, metabolic alternations and immune response. Lung metastasis can be mediated by CSCs such as CD44^hi^ CD36^+^ cancer cells, which favor lipid uptake and metabolism in breast cancer and melanoma. Clinical data have shown that the presence of metastasis-initiating cells positive for CD36, a fatty acid translocase, correlates with a poor prognosis for numerous types of carcinomas.^114^ The two major biomass production (anaplerosis) pathways involved in cellular proliferation are pyruvate conversion to oxaloacetate via pyruvate (PC) and glutamine conversion to α-ketoglutarate. Cancers often show an organ-specific reliance on either pathway. Study have identified higher PC-dependent anaplerosis in breast cancer lung metastasis compared to primary breast cancers.^115^ Breast cancer cells that infiltrate the lungs can produce tenascin C (TNC), and tumor stroma can also provide a source of TNC. TNC can promote the survival and outgrowth of lung micrometastases by enhancing the expression of stem cell signaling components including musashi homolog 1, which is a positive regulator of Notch-signaling and leucine-rich repeat-containing G protein-coupled receptor 5 (LGR5), a target gene of the WNT pathway.^116^ Secretome analysis also identified cancer-specific lung metastasis secretome signatures, such as Nidogen 1 (NID1) which is associated with poor clinical outcomes.^117^ NID1 promotes lung metastasis of breast cancer by increasing cancer cell mobility and promoting adhesion of cancer cells to the endothelium, thereby disrupting its integrity, and promoting angiogenesis.^117^ RARRES3, recently characterized as a lung metastasis suppressor, regulates cancer cell adhesion and differentiation.^118^ B7x, also termed B7H4 or B7S1, is an inhibitory member of the B7 family of T cell co-stimulation, whose expression level is upregulated in metastatic cancers and is associated with lung metastasis of breast cancer. By using B7x knockout mice, Zhang et al. found that host B7x enables cancer cells evade local immunosuppressive responses by interacting with the innate and adaptive immune systems, including tumor associated neutrophils, machrophages and regulatory T cells.^119^

### Inhibitory role of lung host tissue

**Fig. 4:**
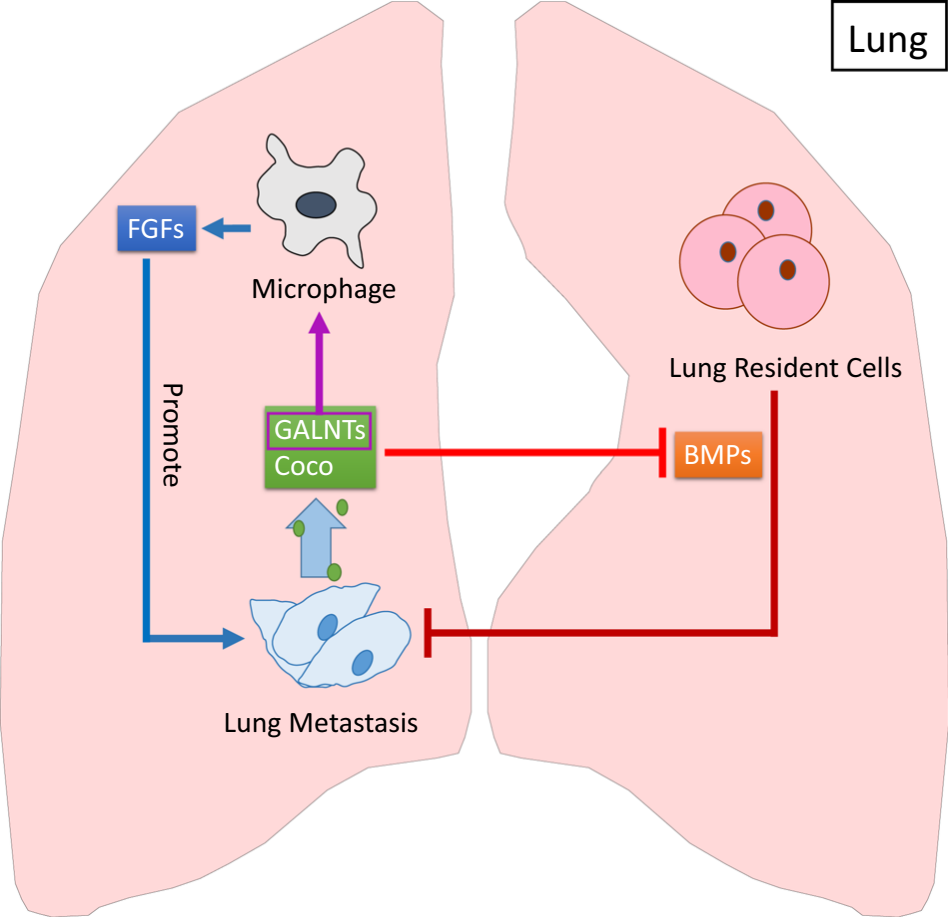
Lung metastatic cancer cells overcome the inhibition of lung cell-derived BMPs. BMPs secreted by lung resident cells can inhibit tumor growth by turning tumor cells into a dormancy state. Cancer cell-derived Coco and GALNTs can inhibit BMPs and reactivates dormant cancer cells to outgrowth in the lung.^120^ GALNTs support metastasis outgrowth by inducing macrophage infiltration and exploiting macrophage-derived FGFs^121^

Lung-derived bone morphogenetic proteins (BMPs) act as anti-metastatic signals in the lung, and lung metastatic breast cancer cells need to overcome their inhibitory effect to form metastasis (Fig. 4). There are several molecules that have this ability and are considered to be metastasis promoters. A gain-of-function cDNA screen reveals that Coco reactivates dormant breast cancer cells to grow in the lung by suppressing the BMPs-mediated CSCs properties inhibition.^120^ One of the polypeptides, N-acetyl-galactosaminyltransferase (GALNT), inhibits BMPs and therefore facilitates lung metastasis initiation by modulating self-renewal properties of CSCs. Elevated by KRAS-PI3K-c-JUN signaling, GALNT14 also induces tumor-promoting macrophage infiltration and exploits macrophage-derived fibroblast growth factors (FGFs).^121^ Moreover, GALNT14 serves as a prognostic marker for the pulmonary relapse in breast cancer patients.^122^

## Liver metastasis

The liver is the most prevalent metastatic sites for all solid cancers and represents the second most common site for breast cancer.^12^ Liver metastases are often larger and more numerous than those of lung metastases, suggesting a metastasis-favorable microenvironment in the liver.^123^ Liver metastasis development in breast cancer patients is associated with stemness and proliferation signaling, such as beta-catenin-independent WNT signaling and Ki67, and confers a poor prognosis.^12, 124^ Liver relapse is associated with ER expression, luminal B subtype, and is prognostic for an inferior post-relapse survival.^125^

### Liver metastasis molecular features

**Fig. 5:**
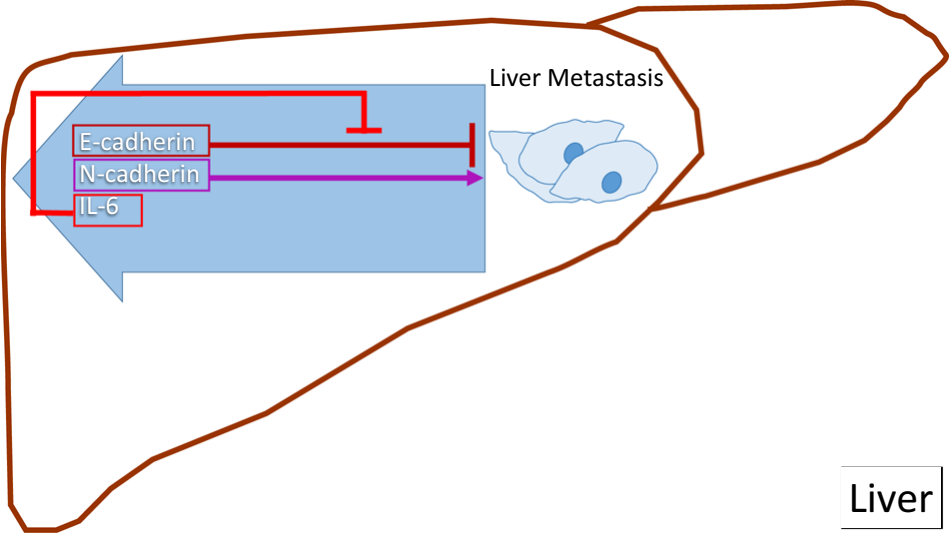
Dysregulation of cell adhesion molecules N-cadherin and E-cadherin in liver metastasis. N-cadherin promotes motility, invasion, and metastasis.^127^ E-cadherin suppresses liver metastasis formation, while high IL-6 levels in breast cancer liver metastases inhibit function of E-cadherin^128^

Breast tumor cell-secreted cytokines and chemokine receptors are associated with liver metastasis. CXCR4 is the most common chemokine receptor mediating liver metastases initiation and CXCR4/CXCL12 participate in extravasation of tumor cells within the liver in a rat model.^126^ Cytokines also stimulate macrophages to produce TNFα, which up-regulates E-selectin expression, and therefore promotes cell adherence to endothelium. Moreover, dysregulation of cell adhesion molecules N-cadherin and E-cadherin contribute to breast cancer liver metastases (Fig. 5). Breast cancer cells with the high levels of N-cadherin enhance liver metastases due to N-cadherin-promoted motility and invasion.^127^ Breast cancer liver metastases maintain high levels of IL6, which decreases the metastasis-inhibitory E-cadherin levels.^128^

Integrin complexes are also involved in breast cancer liver metastasis. The α2β1 integrin complex interacts with the reticular collagen I-rich fibers in liver stroma and inhibition of α2β1 blocks the direct interactions of tumor cells with distinct matrix components and reduces liver metastasis.^129^ Claudin-2 facilitates cell/matrix interactions by increasing the cell surface expression of integrin complexes α2β1 and α5β1 in breast cancer cells.^130^ Although Claudin-2 is weakly expressed in primary breast cancer cells, it is detected in all liver metastases samples, facilitating interactions between tumor cells and primary hepatocytes.^130^ Claudin-2 expression level in liver metastasis is elevated by pan-inhibition of Src family kinase (c) signaling pathways.^131^ Neutralizing antibodies targeting α5β1 or α2β1 can block Claudin-2-mediated adhesion to fibronectin and type IV collagen, and reduce the ability of breast cancer cells to metastasize to the liver.^132^ Therefore, α2β1 or α5β1 complex can promote breast cancer cells metastasize to the liver in the Claudin-2 signaling pathway.^133^

The transmembrane adapter protein DNAX-activating protein of 12 kD (DAP12) can activate multiple signals for several arrays of receptors.^134^ DAP12 expression in breast cancer cells is correlated with a higher rate of bone and liver metastases as well as poor prognosis.^135^ Liver-specific homing of breast cancer cells displays unique transcriptional fingerprints, characterized by downregulation of ECM (stromal) genes.^125^ PPFIA1 (liprin-α1) expression can be significantly higher in the liver metastases than the primary tumors and serves as a potential poor prognostic indicator of increased metastatic relapse in ER^+^/N^−^ (nodal negative) breast cancer group.^136^ β-catenin-independent WNT signaling coincides with a poor prognosis in patients with breast cancer liver metastasis.^124^

Liver-metastatic breast cancer cells exhibit a unique metabolic program compared to bone or lung metastatic cells, characterized by increased conversion of glucose-derived pyruvate into lactate and decreased mitochondrial metabolism.^137^ Pyruvate dehydrogenase kinase-1 (PDK1)-dependent metabolic reprogramming is a key regulation of metabolism and liver metastasis in breast cancer. PDK1 is specifically required for metabolic adaptation to nutrient limitation and hypoxia as a HIF1α target in liver metastatic cells.^138^ Additionally, HIF-regulated genes LOX, OPN, VEGF, and TWIST coordinate to promote breast cancer liver metastasis.^139^ LOX inhibition has no significant effects on primary tumor growth but significantly decreases lung metastases and depletes liver metastases.^139^ The quinoxaline di-N-oxide DCQ blocks breast cancer metastases by targeting the HIF1 pathway and exhibits robust antitumor activity, enhances animal survival, and reduces metastatic dissemination to the lungs and liver.^140^

### Special feature of liver metastasis based on liver biological structure

Liver is a densely vascularized organ with unique biological structure. It has fenestrated vasculatures, and the endothelium without organized sub-endothelial basement membrane. This structure allows the transportation of big molecules, and influences the interactions between cancer cells and liver microenvironment.^133^ Liver metastases can develop the suitable environment for their own growth by replacing the hepatocytes and co-opting the vasculature. However, in contrast to colorectal cancer liver metastases, which expand with concomitant hypoxia-driven angiogenesis, breast cancer liver metastases can grow without hypoxia and angiogenesis.^141^ By using two-photon microscopy, Martin et al.^142^ examined the interaction between cancer cells and the microenvironment during early stage of breast cancer metastases and compared tumor cells in the liver and the lungs. They demonstrated that more tumor cells extravasate to the liver (56%) than the lungs (22%) 24 h after tumor cell injection. There were two subsets of lesions: a majority of lesions remained the same size, consisting of a few cells between days 5 and 12 after injection, which may not utilize the blood supply and remain dormant in the liver. Another subset formed with a patent vasculature formation that have the capacity to establish a small micrometastatic lesion in the liver microenvironment.^142^ This suggests that the same breast cancer cells can show different behavior in different host microenvironment.

## Lymph-node metastasis

Lymph-node metastasis indicates a high risk of distant metastasis. Absence of lymph-node metastases correlates with low risk of distant metastasis, whereas the presence of more than four lymph-node metastases predicts very high risk of distant metastasis.^143^ It has been well known that tumor metastasis to distant sites does not occur exclusively through the axillary lymph nodes (ALD), but also through blood circulation. Therefore, the lymph-node metastatic status should be used as an indicator of the tumor cells’ ability to metastasize. A correlation has been found between tumor size and the percentage of positive lymph-node metastases.^144^

Luminal A, luminal B, luminal-HER2 and HER2-enriched subtypes of breast cancer are highly correlated with lymph-node metastases and poor outcome in the patients with ALD metastases, but not in the patients with tumor-negative lymph-nodes.^145^ A high ratio of lymphovascular invasion and high expression of Ki67 are independently predictive of ALD metastases.^146^ Another potential biomarker, cytoplasmic chromosome segregation 1 Like is significantly associated with ALD metastases although it appears to have no regulatory effects on ALD metastases.^147^

Axillary lymph-node dissection used to be a standard surgical procedure for breast cancer since the 1800s, but has been replaced by sentinel lymph-node (SLN) biopsy, which has become the routine procedure for early breast cancer patients because of its benefits and minor side effects.^148^ When the axillary SLN has no evidence of micrometastases, the nonsentinel lymph-nodes (NSLNs) are unlikely to have metastases.^149^ Comparing to the NSLN-negative group, four kallikrein (KLK) subfamily members (KLK10, KLK11, KLK12, and KLK13) are up-regulated, while B cell antigen receptor (BCR) signaling pathway is downregulated in the NSLN-positive group.^150^ Consistently, breast cancer tissues show a higher expression of KLK10 and KLK11 than the non-carcinoma mammary glands^151^ and the dysregulation of KLK gene family is closely associated with endocrine-related cancer, such as prostate, breast, and ovarian cancers.^152^ Therefore, more studies are needed to confirm the role of KLK family in lymph node metastasis. It is known that the BCR signaling pathway is critical for B lymphocytes development and survival, and plays significant roles in chronic lymphocytic leukemia.^153^ However, this is the first report about the role of BCR signaling in breast cancer lymph node metastasis, which warrants further investigation.

## Conclusion and perspectives

Despite the significant progress made over the past decade with combination of clinical profiling data and experimental models, our understanding of metastasis remains limited. Genetic changes, stemness and signaling pathways influence metastatic progression. Some of these factors universally affect the tumor cells’ capacity for dissemination and colonization, some of them are site-specific to regulate the cross talk between tumor cells and host cells. Comprehensive and integrated analyses at DNA, RNA, and protein levels are expected to reveal the additional mechanisms of cancer metastases. Availability of large open-access knowledge-based database such as Cancer Genome Atlas and the Human Protein Atlas, and analysis from integrative sequence of metastatic cancers will provide new insights about cancer metastasis.

Facing the future challenges of precision medicine, multiple molecular abnormalities of breast cancer have been identified by using targeted sequencing, whole-exome sequencing, RNA sequencing, gene expression analysis, phosphoprotein detection, SNP arrays and ctDNA sequencing which have been also used in clinical trials.^154^ A large numbers of candidate targets have been identified based on genetic screening. However, only a few of them have been validated in clinical studies, such as mutations of phosphatidylinositol-4,5-bisphosphate 3-kinase catalytic subunit alpha (PIK3CA), AKT1and ERBB2, as well as amplifications of fibroblast growth factor receptor 1 and EGFR.^155^ These candidates have shown objective responses in phase I/II clinical trials, suggesting they could be the promising therapeutic targets in breast cancer.^156^ Besides routine molecular genotyping of tumors, large precision medicine-based clinical trials have also been launched to match targeted therapy to the molecular alteration discovered in tumors. For example, studies have evaluated the potentially targetable genomic alterations in breast cancer, and showed that 84% of breast cancers contains at least one genomic alteration, which can be considered as treatment target.^157^ Therefore, combination of predictive value of genomic alterations with clinical relevance is critically important for further progress in this field.

Genomic and genetic studies assist potential applications of immunotherapies. A recent study identified essential genes for cancer immunotherapy by using a genome-scale CRISPR-Cas9 library, and profiled genes whose loss in tumor cells impair the effector function of CD8^+^ T cells. Loss of essential genes for the effector function of CD8^+^ T cells has been linked to the resistance or non-responsiveness of cancer to immunotherapies.^158^ In addition to the molecular biomarkers associated with sensitivity and resistance to immunotherapies in cancer patients with brain metastases, clinically actionable mutations presented in brain metastases can be used for targeted therapies.^159^ Immunotherapy has been ineffective in patients with brain tumors and brain metastases.^160^ However, precision medicine based immunotherapy and targeted therapy bring a promising results for the treatment of brain metastases in lung cancer study.^161^

While metastatic cancers share key mutations with the primary tumors from which they arise, they often develop new mutations as they evolve during metastasis and treatment.^162^ Therefore, real-time analysis and targeted therapy for metastatic tumor rather than archival materials of their primary tumor will be preferable for efficient therapy. Analysis of liquid biopsies including CTCs, cell-free nucleic acid, or extracellular vesicles such as exosomes have gained more attention for precision medicine in the past few years. Liquid biopsies provide a non-invasive way of longitudinally evaluating patient’s outcome, new mutations and response to the treatment, and offer physicians an opportunity to quickly, appropriately adjust to more targeted and efficient treatments. Integrating analysis of liquid biopsies with our understanding of metastatic organotropism will improve future precision medicine for metastatic disease.
